# Open Surgery for Localized RCC

**DOI:** 10.1100/tsw.2007.142

**Published:** 2007-01-22

**Authors:** Kathy Vander Eeckt, Steven Joniau, Hein Van Poppel

**Affiliations:** Department of Urology, University Hospitals Leuven, Belgium

**Keywords:** renal cell carcinoma, open surgery, nephron-sparing surgery

## Abstract

The only possibility for cure in localized renal cell carcinoma (RCC) is surgery. Open radical nephrectomy (RN), as described by Robson, has long been the gold standard. Nevertheless, as a consequence of the increased use of abdominal imaging modalities, a continuing stage migration towards small, low-grade RCC lesions has become evident during the last decades. Together with this stage migration, nephron-sparing surgery (NSS), less-invasive therapies (laparoscopic RN and NSS), and minimally invasive therapies (radiofrequency ablation [RFA], cryoablation) have been developed and are gaining popularity. The value of laparoscopic RN and open NSS are acknowledged worldwide, but the value of laparoscopic NSS, RFA, and cryoablation remains to be established. Despite this evolution, there is still a place for open surgery for localized RCC. Open NSS is, at present, considered the standard of care for localized RCC less than 4 cm, while open RN still has a place for larger lesions, certainly when an extended lymph node dissection or adrenalectomy is warranted, or when a tumor thrombus is extending into the inferior vena cava. This review provides the data that support open surgery in clear, selected cases of RCC.

## INTRODUCTION

On a global scale, over 200,000 new cases of kidney cancer are diagnosed annually and more than 100,000 deaths occur from this disease[1]. Renal cell carcinoma (RCC) accounts for 3% of all adult malignancies and increases at a rate of 2.5% a year. This increase in RCC could partially be explained by the increased detection of asymptomatic, incidental tumors through the widespread use of noninvasive imaging techniques (ultrasonography, computed tomography [CT], and magnetic imaging). Kidney cancer is the most lethal of urologic cancers. More than 40% of the patients with RCC will develop metastases[2].

Although Robson introduced the radical nephrectomy (RN) as the standard treatment for localized RCC in patients with a normal contralateral kidney in 1963, other potential therapeutic options arose as a result of the increased number of small tumors and the advances in surgical technology[3]. In an attempt to minimize operative morbidity, less invasive options like laparoscopic radical and partial nephrectomy, and minimally invasive options like cryoablation, radiofrequency ablation (RFA), and high-intensity focused ultrasound (HIFU) have been developed. Despite the trend towards these less-invasive new approaches, a number of indications for continuing open surgical resection remain in the management of RCC.

## RADICAL NEPHRECTOMY

Robson's retrospective analysis of 88 RN is considered the most influential paper on RCC published in the urologic literature[3,4]. The fundamentals of open RN included early ligation of the renal vessels to minimize the risk of hematogenous spread of vascular tumor emboli, en bloc excision of Gerota's fascia including the kidney and adrenal gland, and an extensive lymphadenectomy (para-aortic and paracaval nodes form the crus of the diaphragm to the bifurcation of the aorta). This approach accomplished several objectives: first, the removal of the ipsilateral adrenal gland, second the excision of lymphatic metastases, and finally, accomplishment of a more adequate margin than could be achieved by a simple nephrectomy. With this approach, Robson achieved an improved overall 5-year survival rate of 52%[3]. Contemporary cohorts show a 5-year survival rate of 75–96, 63–95, 38–76, and 11–32% for, respectively, T1, T2, T3, and T4 tumors (Table 1).

**TABLE 1**
**Five-Year Survival Rate of RN by TNM Stage**

| | No. of Patients | TNM Stage (%) | | | |
|---|---|---|---|---|---|
| | | T1 | T2 | T3 | T4 |
| Guinan et al.[5] | 2473 | 75 | 63 | 38 | 11 |
| Kinouchi et al.[6] | 328 | 96 | 95 | 70 | 24 |
| Javidan et al.[7] | 381 | 95 | 88 | 59 | 20 |
| Tsui et al. [8] | 643 | 91 | 74 | 76 | 32 |
| Gettmann et al.[9] | 1547 | 95 | 80 | 56 | 20 |

Since the publication by Robson, open RN was considered the gold standard against which all newer approaches (laparoscopic RN, partial nephrectomy, laparoscopic partial nephrectomy, and ablative techniques) should be compared. However, the role of adrenalectomy, extended lymph node resection, and complete removal of the kidney are debated in a number of indications. The increased use of imaging modalities (abdominal ultrasound and CT) has led to the detection of early-stage, low-grade tumors. These tumors have a low incidence of adrenal and lymphatic involvement, rendering the need for such radical resection questionable.

## ADRENALECTOMY

Years ago, it was suggested and more recently argued again that removing the ipsilateral adrenal gland is not always necessary in the absence of radiographic adrenal enlargement, unless the malignancy either extensively involves the kidney or is located in the upper pole of the kidney[8]. The debatable issue of ipsilateral adrenalectomy is even more relevant in patients undergoing nephron-sparing surgery (NSS), patients with bilateral tumors, and tumors in solitary kidney with contralateral adrenal absence or adrenal abnormalities. The complete loss of adrenal function in such cases would require chronic glucocorticoid and mineralocorticoid replacement.

In order to decide if routine ipsilateral adrenalectomy is necessary, a few unresolved questions need to be answered.

The first and most important question is the *incidence of adrenal involvement* by RCC. Previous studies noted malignant involvement of the ipsilateral gland in less than 10%. Recent reports demonstrated a lower incidence between 2.7 and 5.7% (Table 2).

**TABLE 2**
**Recommendations for Adrenalectomy in Several Recent Studies**

| | No. of Patients | Adrenal Metastases (%) | Recommendation |
|---|---|---|---|
| Tsui et al.[10] | 511 | 5.7 | Risk factors (high stage, upper pole, multifocality, renal vein thrombus) |
| Paul et al.[11] | 866 | 3.1 | Preoperative examinations or algorithms |
| Sawai et al.[12] | 229 | 2.7 | Abnormal CT; large tumors |
| Kuczyk et al.[13] | 819 | 3.3 | Abnormal CT |
| Moudouni et al.[14] | 268 | 5 | Abnormal CT; large, upper-pole extracapsular lesions |
| Autorino et al.[15] | 192 | 4.1 | Abnormal CT; large, high-risk tumors |
| De Sio et al.[16] | 201 | 4.4 | Abnormal CT; large, high-risk tumors |
| Siemer et al.[17] | 1010 | 5.5 | Tumor > 4 cm, or T3-4 |
| Yokoyama et al.[18] | 247 | 2.8 | Abnormal CT; large tumors |

The second issue is the *survival benefit* of adrenal preservation. Older studies reported that the preservation of the ipsilateral adrenal does not influence the tumor recurrence and survival rate in comparison to standard RN, but those studies may be biased by the fact that adrenal involvement was almost always associated with high-stage disease[16]. Recent studies stated that only a few patients who have solitary, mainly microscopic, ipsilateral adrenal metastases may benefit from adrenalectomy. The remaining patients with adrenal involvement have disseminated RCC with a poor prognosis due to the other metastases. Paul et al.[11] stated that only 0.6% had solitary adrenal metastases. The mean survival of these patients, patients with distant metatases, and with metastases, was 21, 84, and 11 months, respectively.

De Sio et al.[16] reported two solitary adrenal metastases in 201 patients (1%) who had a mean disease-free survival time of 69 months. The seven other patients with adrenal involvement had disseminated metastases with a mean disease-specific survival time of only 24 months. Autorino et al.[15] also reported only two of 192 patients (1%) with a better disease-free survival time. All these studies show that the prognosis is poor for RCC with ipsilateral involvement, even with complete removal[10,11,12,13,14,15,16,17,18].

A *definition of the clinical and pathological factors that may predict adrenal involvement* is the third issue. Several studies demonstrated a correlation between left-sided, large tumors, tumors in the upper pole, advanced T stage, and adrenal involvement (Table 3)[10,12,15,16].

In a study of 511 patients undergoing RN with ipsilateral adrenalectomy, Tsui et al. demonstrated that CT missed microscopic adrenal involvement in three patients only. Others suggest that normal adrenal images on CT exclude adrenal involvement[10,12,14,16,17]. In recent studies, preoperative CT demonstrated an 87–100% sensitivity and a 76–99.6% specificity for adrenal metastases [10,12,13,14,15,16].

In conclusion, strict indications for ipsilateral adrenalectomy are a preoperative CT showing adrenal lesions or RCC with increased risk factors, such as high-stage, upper-pole location; multifocality; and renal vein thrombus.

**TABLE 3**
**Incidence of Adrenal Metastasis in T1-2 and T3-4 Tumors**

| | No. of Patients | Adrenal Metastases (%) | T1-2 | T3-4 |
|---|---|---|---|---|
| Tsui et al.[10] | 511 | 5.7 | 0.6 | 8.1 |
| Paul et al.[11] | 866 | 3.1 | 0.8 | 10.1 |
| Moudouni et al.[14] | 268 | 5 | 0.9 | 13.4 |
| Autorino et al.[15] | 192 | 4.1 | 1.3 | 5.8 |
| De Sio et al.[16] | 201 | 4.4 | 1.3 | 6.4 |

## LYMPH NODE DISSECTION

The role of lymph node dissection (LND) in the surgical management of RCC remains an area of debate. The overall risk of lymph node metastases with RCC is approximately 20% and it is known that life expectancy decreases considerably when lymph nodes are present, with 5-year overall survival rates between 14–20%[19,20]. The risk of lymph node metastases depends on the primary tumor stage, size, and presence of systemic metastasis. It is reported that node-positive status is associated with larger, higher-grade, and locally advanced tumors[19]. Thus, we need to make a difference between localized disease, lymph node invasion only, or advanced metastatic disease.

In patients with RCC confined to the kidney on imaging (N0M0), LND can improve staging accuracy by identifying nodal metastases that were not suspected on preoperative imaging. Proponents of LND put the safety of the procedure forward as an argument to perform an LND systematically[19,20,21]. In a recent EORTC Phase III trial, Blom et al. have compared RN with and without extended LND in clinically N0M0 RCC. Only 3% of nodal disease was not identified preoperatively and only 1% was not palpable during surgery[21]. Thus, LND allows a more accurate pathological staging and, theoretically, a few patients might benefit from prophylactic LND in the case of microscopic lymph node involvement. Nevertheless, we must recognize that, to date, no efficacious adjuvant therapy has been identified in the case of lymph node involvement and the exact therapeutic value of LND remains controversial[22]. Recent studies proved that the overall, local, and distant recurrence-free survival of the patients with clinically node-negative disease who underwent no LND was the same as the survival of those with node-negative disease with LND. And survival was independent of the extent of LND[19,21,23,24].

In patients with RCC and positive lymph nodes only (N+, M0), the only purpose of LND is total resection of the disease and cure. Although the number of patients with N+M0 is small, these are the patients who have a chance to be rendered free of disease with LND. Recent studies reported a significant survival advantage in patients with node-positive disease who underwent LND compared with those who did not undergo LND[19,25]. Another argument in favor of LND is that up to 30% of the patients might have inflammatory nodal enlargement only, even if the lymph nodes are enlarged on imaging[26].

Patients with metastatic RCC and lymph node involvement (N+, M+) have a significantly worse 5-year survival rate than patients with metastatic disease only[20]. Pantuck et al. reviewed 900 patients among whom 86 were N+M+. Patients with lymph node involvement had poorer response to immunotherapy and those patients who had LND for node-positive disease had a better response to immunotherapy and higher survival rate than those with nephrectomy only[19]. Despite the positive impact of LND on survival, there was no difference in local or systemic recurrence rate. Vaselli et al. investigated 154 patients with distant metastasis and cytoreductive nephrectomy. They found that the presence of positive lymph nodes was associated with shorter survival. Those with complete LND showed a better survival than those without LND[20].

No standard rules for LND exist, but taking into consideration the results of the above-mentioned recent studies, some recommendations can be made. First, routine use of regional LND can be omitted with patients with no suspicion of lymph nodes before or during surgery. Second, if the surgeon suspects positive lymph nodes during surgery in patients without advanced metastasis, LND can be performed in order to achieve accurate staging and to provide a chance for cure in selected patients. Third, although efficient adjuvant therapy is still not available, LND seems to provide a survival advantage in cytoreductive surgery.

## OPEN PARTIAL NEPHRECTOMY

Although RN, as described by Robson et al., has been the gold standard operation for patients with RCC for many decades, there is a trend towards NSS[3,27]. This evolution is the result of improved surgical technique, standardized staging, advanced radiological imaging, and downward migration toward the diagnoses of asymptomatic, incidental, smaller, lower-stage lesions, and the associated low rate of adrenal and lymph node metastases[28]. The TNM classification has recently been adapted to differentiate between tumors measuring ≤ 4 cm (T1a) and 4–7 cm (T1b)[29].

The goal of NSS is, first, oncological complete local excision of the tumor with minimal technical complications and, second, optimal functional preservation of the renal remnant.

The initial indications of NSS, absolute and relative indications, are widely accepted. Absolute indications involve patients in whom RN would render the patient anephric, with the need for immediate renal replacement therapies. More specifically, this concerns patients with an anatomically or functionally solitary kidney due to unilateral renal agenesis, previous contralateral nephrectomy, bilateral RCC, or irreversible impairment of contralateral renal function from a benign disorder. Relative indications are those in which the contralateral kidney has pre-existing renal disease or its future function is threatened. The former conditions include calculous disease; chronic pyelonephritis; renal artery stenosis; vesicoureteral reflux with or without renal scarring; chronic renal obstruction from congenital or acquired causes or systemic diseases, such as diabetes, hypertension, and nephrosclerosis; and patients with known tumor multifocality, such as patients with a papillary histology and hereditary forms of RCC (e.g., Von Hippel-Lindau).

Elective indications are more controversial. These include patients with small, localized, often incidental tumors and a normal contralateral kidney. There are several reasons to expand the indications of NSS to this patient group.

First, several recent studies demonstrate a survival rate and a recurrence rate in NSS comparable to RN for low-grade, low-stage RCC of 4 cm or smaller (Table 4). Although oncological results seem to be equal for NSS and RN (Table 5) for small tumors, and the advantage in renal function is evident, NSS is still underused, as shown in a recent analysis[42]. NSS for tumors less than 4 cm (T1a) is widely accepted at present even for elective indications, while the use of NSS for patients with larger T1 tumors (T1b) is much more controversial (Table 6). Hafez et al. studied the impact of tumor size in 485 patients who underwent NSS. They demonstrated significantly better survival and lower local recurrence rates for tumors less than 4 cm compared to those larger than 4 cm[32]. However, recent studies suggest that NSS can be effectively and safely used to treat patients with tumors up to 7 cm. Nevertheless, they almost all reported a higher local recurrence rate than in RN[37,39,40]. More data and long-term results are needed before elective NSS can be generally expanded to RCC larger than 4 cm.

Second, the detection rate of small solid renal masses is increasing because of better imaging. Radiological imaging is nearly always diagnostic in large tumors, but smaller masses are more difficult to characterize. Definitive pathological examination has revealed that 12–22% of masses smaller than 4 cm are benign[33,36,39,43]. In these patients, the classical approach would be RN. NSS would save them from an unnecessary nephrectomy. Theoretically, fine needle aspiration (FNA) could offer a solution for small masses. However, Van Poppel et al. reported that FNA is not recommended if conservative surgery is planned, unless renal involvement by metastasis or lymphoma is suspected because the low sensitivity of FNA and the major complications[28].

Third, the main perceived benefit of NSS *in absolute and relative indications* is preservation of maximum renal parenchyma to avoid end-stage renal disease (Table 3), although this still has to remain in balance with an adequate cancer control. Several studies showed that in patients who require NSS, this procedure can safely preserve renal function and effectively treat malignancy (negative surgical margins, no local recurrence)[44,45,46]. On the other hand, the long-term functional advantage of NSS remains to be proven definitely *in elective conditions*. The rationale of NSS in patients with a normal contralateral kidney and a normal renal function is to preserve as much renal parenchyma as possible and to prevent chronic renal insufficiency. Data from long-term follow-up after unilateral donor nephrectomy for transplant donation show a minimal disadvantage to the renal function. Because the donor population, selected to minimize comorbidities known to increase the risk of renal insufficiency, differs from the patients with renal cancer, further investigation was necessary. McKiernan et al. compared the development of chronic renal insufficiency after partial and radical nephrectomy in 290 patients with a renal tumor of 4 cm or less and a normal contralateral kidney[35]. Lau et al. reported the long-term follow-up of a matched comparison of RN and NSS in 328 patients (164 patients in each group) with a small unilateral tumor and a normal kidney. Both studies concluded that patients undergoing RN are at a greater risk of chronic renal insufficiency[34]. Although the advantage of NSS for the renal function is promising, further study is required. These two studies are in contradiction to what was believed from donor nephrectomy studies.

**TABLE 4**
**Recent Studies on the Outcome of Patients Undergoing NSS for Localized RCC**

| | Ind* | No. of Patients | Tumor Size (cm) | Disease-Free Survival (5 Years) % | Local Recurrence (%) | Median Follow-Up (Months) |
|---|---|---|---|---|---|---|
| Van Poppel et al.[30] | A | 76 | 0.9–15 | 96 | 0 | 75 |
| Herr[31] | E | 70 | 1.1–6.5 | 97 | 1.5 | 120 |
| Hafez et al.[32] | A | 310 | <4 | 96 | 0.6 | 47 |
| | | 175 | >4 | 86 | 0.8 | 47 |
| Lee et al.[33] | A | 79 | 0.5–4 | 100 | 0 | 40 |
| Lau et al.[34] | E | 164 | 3.3 ± 1.7 | 98 | 1 | 44 |
| Mc Kiernan et al.[35] | E | 117 | 0.9–4.0 | 100 | 1.2 | 25 |
| Kural et al.[36] | E | 50 | 3.63 ± 1.36 | 100 | 0 | 33.1 |
| Patard et al. [37] | A | 314 | 0–4.0 | 97.8 | 0.8 | 51 |
| | | 65 | 4.0–7.0 | 93.8 | 3.6 | 51 |
| Leibovich et al.[38] | A | 91 | 4.0–7.0 | 98 | 5.4 | 64 |
| Becker et al.[39] | E | 69 | 4.1–10.0 | 100 | 5.8 | 70 |
| Mitchell et al.[40] | A | 66 | 4–15.0 | 93.5 | 2.3 | 34 |

- Ind = indication, E = elective, A = all indications.

Fourth, the risk of contralateral tumor recurrence has been used as an argument for a conservative approach in patients with RCC and a normal contralateral kidney[31,45]. Previous studies noted an incidence of contralateral recurrence between 1 and 5%[48,49,50]. Positive margins and multifocality were significant predictors for contralateral tumor recurrence in clear cell RCC, while nuclear grade was a significant predictor in papillary RCC[49]. This contralateral recurrence may be low, but because it is unpredictable, it seems significant enough to support NSS for small tumors.

**TABLE 5**
**Recent Studies Comparing Cancer-Specific Survival in Radical vs. NSS**

| | No. of Patients | | 5-Year Disease-Free Survival (%) | | Local Recurrence-Free Survival (%) | | Median Follow-Up (Months) |
|---|---|---|---|---|---|---|---|
| | RN | NSS | RN | NSS | RN | NSS | |
| Lerner et al.[41] | 209 | 185 | 89 | 89 | — | 94.4 | 44 |
| Lau et al.[34] | 164 | 164 | 97 | 98 | 97 | 99 | 44 |
| Lee et al.[33] | 183 | 79 | 96 | 96 | 100 | 100 | 40 |
| Mc Kiernan et al.[35] | 173 | 117 | 100 | 100 | 98.6 | 96.4 | 25 |
| Leibovich et al.[38] | 841 | 91 | 86 | 98 | 97.7 | 94.6 | 60 |
| Patard et al.[37] | 1075 | 379 | 97.4 | 97.8 | 99.6 | 99.8 | 51 |
| Becker et al.[39] | 369 | 241 | 95.5 | 97.8 | — | 94.4 | 64 |
| Mitchell et al.[40] | 66 | 33 | 97.8 | 96.2 | 98.8 | 97.7 | 60 |

**TABLE 6**
**Recent Studies on the Oncological Outcome and Analysis of Renal Function after NSS**

| | No. of Patients | Disease-Free Survival (5-Years) (%) | Local Recurrence (%) | Creat. Preop. (mg/dl) | Creat. Postop. (mg/dl) | No. of Patients End-Stage Renal Disease (%) |
|---|---|---|---|---|---|---|
| **Absolute indications** | | | | | | |
| Fergany et al.[44] | 96 | 88 | 4 | 1.4 ± 0.5* | 1.8 ± 0.8* | 7,3 |
| Adkins et al.[45] | 30 | — | 0 | 1.5** | 1.8** | 10 |
| Saranchuk et al.[46] | 54 | 88 | 6 | 1.4** | 1.5** | 3,7 |
| Fergany et al.[47] | 400 | 89 | 3.5 | 1.4 ± 0.5* | 2.2 ± 1.3* | 5 |
| **Elective indications** | | | | | | |
| McKiernan et al.[35] | 117 | 100 | 1.2 | 0.98* | 1.0* | 0 |
| Lau et al.[31] | 164 | 96 | 1 | 1.1** | 1.2** | 1,2 |

* Mean creatinin preop/mean creatinin after 10 years of follow-up.

** Median creatinin preop/median creatinin after median 24 months of follow-up.

And last, studies have shown that patients with more normal functioning renal tissue (NSS) have a better quality of life (constipation, fatigue, pain, and sleep disturbance) than those with less (RN)[51,52,53]. Comparing hospital costs and mean hospital stay for NSS and RN resulted in equivalent direct hospital costs and length of stay[54].

Over the years, complications of NSS have been well characterized and include early complications like intraoperative death, perioperative death, hemorrhage, acute renal failure, sepsis, dialysis, infection, urinary fistula; and late complications like chronic renal failure, proteinuria, wound herniation, and chronic haemodialysis. Thompson et al.[55] investigated the complications of 823 open NSS. They compared the NSS performed between 1985 and 1995 with those performed between 1996 and 2001. They concluded that due to increased experience, improved anatomical imaging, and enhanced perioperative care, the complications decreased significantly in the last years. Even after adjusting the groups for type of surgery (elective vs. imperative), there was a significant decrease[55]. Campbell et al.[56] reported that the complication rates after NSS are related to tumor diameter, with larger tumors having a greater risk of perioperative complications. Recent studies recognize that NSS may confer a greater risk of perioperative complications, but these possible complications have to be weighed against benefits of NSS[34,35,36,37,38,39,40].

A margin of 1 cm of normal-appearing parenchyma was long considered the standard in NSS[57]. However, wide margins may compromise the residual renal function. The optimal resection margin is still debated because satellite lesions can be found more than 1.0 cm beyond the primary tumor[58,59,60]. Sutherland et al.[61] investigated the effects of surgical margin on recurrence. They concluded that the margin width is irrelevant if the tumor is completely resected and that it was not correlated with disease progression. The oncological result was independent of the margin width[61]. Lapini et al. even showed that simple enucleation is a safe and acceptable approach[62].

## RENAL VEIN AND INFERIOR VENA CAVA (IVC) INVOLVEMENT

In patients with RCC, the renal vein and the IVC are involved in 16–33 and 4–13%, respectively[63]. As extensive tumor thrombi can be present without evidence of lymph node involvement and distant metastasis, RN presents an exceptional challenge for the urologist from both the oncological and surgical standpoints. Once, such a phenomenon was thought to be a poor prognostic factor and was considered inoperable because of the major risk involved with surgery. Because of the advances in surgical technique, it became possible to remove the tumor thrombus with significantly reducing surgical risk. Because such tumors require accurate resection with optimum exposure and technical expertise, the open surgical approach is still indispensable.

Accurate definition of the cranial extent of the thrombus is critical in order to determine the surgical approach. The Mayo Clinic defines five different levels of tumor thrombus: level 0 (renal vein only), level I (thrombus extending 2 cm above the renal vein), II (thrombus extending >2 cm above the renal vein, but below the hepatic veins), III (thrombus at the level of or above the hepatic veins, but below the diaphragm), and IV (thrombus extending above the diaphragm)[64].

The natural history of RCC invading the IVC is reported as extremely unfavorable because it is often associated with distant metastasis. Patients presenting with IVC involvement without distant metastases have a higher risk of dying from cancer than patients with renal vein involvement only. In both cases, RN with tumor thrombectomy remains the treatment of choice. This kind of surgical treatment requires multidisciplinary actions (urologist, liver surgeon, cardiac surgeon, and anesthesiologist) because mobilization of the liver is needed in level III thrombi, or cardiopulmonary bypass and hypothermic circulatory arrest in level IV thrombi. Most patients with renal vein and level I and II thrombus can be treated through a subcostal incision. A thoracoabdominal approach is favorable in level III and IV thrombi because of a better exposure and proximal approach in IVC with median sternotomy in level IV thrombi. The primary goal of all surgical techniques must be to take control of the vena cava above the tumor thrombus to avoid embolism.

It is evident that such a multidisciplinary approach leads to considerable perioperative morbidity and mortality. Zisman et al.[65] compared 74 patients with renal vein extension, 87 with IVC extension, and 491 without tumor thrombus who underwent nephrectomy with and without thrombectomy, respectively. Patients with thrombectomy had a higher risk of surgery-related death than those without thrombectomy (3.1 vs. 0.8%). They also reported an overall complication rate of 27.6% in patients with venous involvement. The major intraoperative complications were adjacent organ laceration, arrhythmia, hypertension, and tumor embolism. The most common postoperative complication was bleeding with high transfusion need. Early morbidity was 47.5% due to urinary and pulmonary infections, hematomas in the renal fossa, embolism, splenectomy, and respiratory failure[65]. Perioperative mortality is between 3.1 and 8%[65,66,67].

Patients with venous invasion had significantly shorter survival than those without venous invasion[63,68]. Overall 5-year survival rate of RCC with venous invasion and M0 is reported to be between 34 and 69%[63,66,67,69,70]. Most studies reported that there was no survival difference based on the proximal extent of the tumor thrombus[63,65,66,67,69,70]. Nevertheless, Zisman et al. reported contradictory results in a study of 264 patients with tumor thrombus extension; disease-specific survival decreased as thrombus extent increased and patients with tumor thrombus in the renal vein had better prognosis than those with tumor thrombus in the IVC[65,68,69].

In conclusion, although surgery for RCC with venous involvement leads to a high perioperative mortality and complication rate, the improvement in overall survival in patients with renal vein or IVC involvement after complete surgical removal in the absence of metastatic disease justifies aggressive surgical management.

## CONCLUSIONS

Clear indications for the open surgical approach remain for the treatment of RCC. Open surgery is still an indispensable approach for tumors with venous involvement, positive lymph nodes, or adrenal metastases, or for cytoreductive surgery followed by immunotherapy. For small tumors, smaller than 4 cm, open NSS is still the standard to which all newer, minimal invasive techniques (laparoscopic NSS, cryoablation, HIFU, and RFA) should be compared.
